# Dr. Girdlestone, on Apoplexy

**Published:** 1802-02

**Authors:** T. Girdlestone

**Affiliations:** Yarmouth


### *To the Editors of the Medical and Phyfical Journal.*

GENTLEMEN,

I Have to requeft the favour that you will do me the juftice to give an early infertion in your ufeful Journal to the following letter of Your's, &c.

*Yarmouth, Jan.* 4, 1802. T. GIRDLESTONE, M. D.

---

I beg leave to contradict a report circulated in the Medical Journal of laft month by Dr. Langflow, that Mr. Crowfoot's pamphlet is the joint work of me and Mr. Crowfoot. And alfo to obferve, that I do not find in Mr. Crowfoot's book the fentence which Dr. Langflow has given with inverted commas, as if it had been a real quotation from Mr. Crowfoot's work.

Whether fuch general propofitions on the caufe or cure of every variety of Apoplexy, can be drawn from a fair perufal of Mr. Crowfoot's work, I fhall leave others to determine.

But in page 561, of the 6th volume of the Medical Journal, Dr. Langflow fays,

" For I believe at the prefent moment there is only one opinion on the fubject, except that of Mr. Crowfoot's, that real apoplexy is caufed by compreffion of the brain, and always, when the fcull is not fractured, by effufion of blood or ferum.

In page 77 of the laft Medical Journal, Dr. Langflow fays, " Since the publication of your laft number, a report has been been circulated in this neighbourhood, either from mifconception or fome worfe motive, that in my anfwer to Mr. Crowfoot upon Apoplexy, I have intended to affert that no cafe of apoplexy ever *did* or *can* occur without extravafation of blood upon the brain, &c." And in a note in the fame page, Dr. Langflow fays, "Upon reference to Mr. C's ftatement, I obferve, that he fays upon my return from the fick chamber, &c. &c. to the parlour, that I declared there was *considerable* extravafation of *blood* upon the brain. Now the parties who were prefent at that converfation, affert that they did not hear me ufe the word *considerable*, or *blood*." Yet in p. 561 of the Medical Journal, Dr. Langflow gives his definition of extravafation in the following words. "The term extravafation ought to be ufed, when the blood or fluids efcapes through a rupture in the coats of the veffels."

Thefe paffages are fair quotations from Dr. Langflow's writings; not quotations from extracts which Dr. Langflow might have taken from other authors; not literary forgeries. But, as I had no more fhare in the opinions or compofition of Mr. Crowfoot's work, than Dr. Bradley or Dr. Batty had in the opinions or compofition of Dr. Langflow's publications in the Medical Journal, I do not feel myfelf bound to take any farther part in this controverfy.

